# Interrelationships between migraine over the counter (OTC) painkillers and hypertensive disorders of pregnancy: A protocol for systematic review and meta-analysis

**DOI:** 10.1097/MD.0000000000028049

**Published:** 2021-12-10

**Authors:** Shalati L. Nkuna, Wendy N. Phoswa

**Affiliations:** Department of Life and Consumer Sciences, University of South Africa (UNISA), Science Campus, Private Bag X6, Florida, Roodepoort, South Africa.

**Keywords:** hypertensive disorders of pregnancy, migraine, over the counter pain killers

## Abstract

**Background:**

Migraine during pregnancy is common and has been reported to affect up to 10% of pregnancies. Irrespective of the type of migraine, over the counter (OTC) painkillers are used as a migraine treatment plan. Growing evidence suggests that OTC painkillers have effects on the mother, the child and hypertensive disorders of pregnancy, including hypertension, eclampsia and pre-eclampsia, but inconsistent findings have been reported. The aim of this study is therefore to investigate the association between the use of migraine OTC painkillers and hypertensive disorders during pregnancy.

**Methods:**

Databases such as Pubmed, Cochrane library, ScienceDirect and google scholar will be searched to identify eligible studies. Studies will be included if they are randomised controlled trials, cohort, and matched cohort, and cross-sectional studies of pregnant women with reported use of OTC painkillers to treat migraines at any stage of their pregnancy. The results will be reported based on the preferred reporting items for systematic reviews and meta-analysis 2009 statement, and article screening and selection process will also be demonstrated through a preferred reporting items for systematic reviews and meta-analysis for protocols flow diagram. The data will then be extracted by 1 reviewer and checked by another for accuracy. The quality and risk of bias of eligible studies will be performed by both reviewers using the Hoy tool and grading of recommendations assessment, development and evaluation tool. The data will be analysed using Review Manager 5.3 (RevMan 5.3) software.

**Ethics and dissemination:**

The review and meta-analysis will not require ethical approval and the findings will be published in peer-reviewed journals and presented at local and international conferences. Findings from this study will help to improve knowledge on the understanding of the effects OCT on hypertensive disorders of pregnancy. This study will also provide new information on the management of migraine during pregnancy.

**Systematic review registration:**

International prospective Register of Systematic Reviews (PROSERO) number: CRD42021232232.

## 1 Introduction

During childbearing years, migraine affects about to 25% of females.^[1]^ Migraine in the general population, is a widespread neurological disorder that is characterized by headache with extreme pulsating one-sided attacks, frequently followed by photophobia and nausea that can last up to 72 hours.^[2]^ Headaches can be both primary and secondary during pregnancy, and in the last case, can be a sign of a life-threatening condition.^[3]^ Primary headaches include tension type headache and migraines. While stroke, cerebral venous thrombosis, subarachnoid haemorrhage, pituitary cancer, eclampsia, preeclampsia, idiopathic intracranial hypertension, and reversible cerebral vasoconstriction syndrome are the most common types of secondary headaches. Typically, these painful, throbbing headaches are felt on 1 side of the head and result from blood vessel widening in the brain. Some symptoms of migraines include nausea, vomiting, and sensitivity to light.^[4]^ They have no detrimental effects on the mother or fetus during pregnancy. However, other studies report that migraines are associated with increased risk of both adverse maternal and fetal outcomes, these may include hypertensive disorders of pregnancy (HDP), increased risk of miscarriage, caesarean sections and giving birth to a child with low birth weight.^[5]^

According to Folk (2018),^[6]^ there are mainly 4 categories of HDP namely; chronic hypertension, gestational hypertension, pre-eclampsia, and chronic hypertension with superimposed preeclampsia as recommended by the National High Blood Pressure Education Program Working Group on high blood pressure in pregnancy. These disorders are a major cause of maternal, perinatal morbidity and mortality and affect up to 10% of pregnancies. As pregnant women suffering from migraine stop or switch medications throughout their pregnancy, the type of medications they use, specifically over the counter (OTC) painkillers may have some effects on the HDP.^[7]^ Many of these women switch from triptans and nonsteroidal anti-inflammatory drugs (NSAIDs) to paracetamol, which constitutes most of the analgesic use for treatment of migraine. For the treatment of acute migraine attacks in pregnant women, the following drugs are preferred: paracetamol, NSAIDs and sumatriptan. For chronic migraine, a 100 mg dose of topimarate is considered a safe treatment in pregnancy.^[8]^ Prophylaxis of migraine should be done if patients experience at least 3 prolonged serious attacks per month, which are especially debilitating or unresponsive to symptomatic treatment and are likely to trigger complications. Nonpharmacologic treatments should be preferred, but if they are not successful, low doses of β-blockers and amitriptyline should be used in preventive care.

Although most OTC drugs have an excellent safety profile, some have unproven safety or are known to adversely affect the fetus and may have some detrimental effects on HDP depending on the type of drug, the dose, duration of therapy, the period of gestation, and the time elapsed between maternal administration and delivery. Women who used acetaminophen during the third trimester of pregnancy had an increased risk of preeclampsia.^[9]^ The prenatal use of NSAIDs and aspirin have been reported to increase the risk of miscarriage.^[10]^ NSAIDs given to pregnant women cross the placenta and may cause embryo–fetal and neonatal adverse effects. Increased risks of miscarriage and malformations are associated with NSAID if used in the early stages of pregnancy. Conversely, exposure to NSAIDs after 30 weeks’ gestation is associated with an increased risk of premature closure of the fetal ductus arteriosus and oligohydramnios.^[11]^ Fetal and neonatal adverse effects affecting the brain, kidney, lung, skeleton, gastrointestinal tract, and cardiovascular system have also been reported after prenatal exposure to NSAIDs. However, the administration of NSAIDs to postpartum patients with hypertensive disorders of pregnancy is not associated with a change in blood pressure.^[12]^ The Federal Drug Administration recommends avoiding the use of NSAIDs in pregnancy at 20 weeks or later because they can result in low amniotic fluid as well as other complications for the fetus, including rare kidney problems. Researchers found that, compared to women who did not use NSAIDs, women with pre-eclampsia and extreme predelivery characteristics who used NSAIDs did not have an elevated risk of chronic postpartum hypertension. The results have led researchers to conclude that, for this group of women, NSAIDs are safer than opioids. The overall impact of paracetamol on blood pressure is uncertain. Since paracetamol is frequently proposed as a safer alternative to NSAIDs, more prospective evidence now appears to be required to resolve the impact of paracetamol on blood pressure.^[13]^ Findings on the use of aspirin during pregnancy are contradictory. Some studies report that aspirin initiated before 16 weeks of gestation can reduce the risk of pre-eclampsia and severe pre-eclampsia.^[14]^ In multiple randomized trials, aspirin taken at dosages between 60 and 150 mg/d decreased the incidence of preeclampsia, preterm birth, and intrauterine growth restriction (IUGR) in women who had an elevated risk of pre-eclampsia.^[15]^ However, using aspirin does not reduce the rate at which pre-eclampsia; gestational hypertension; early-onset pre-eclampsia or severe pre-eclampsia can progress during pregnancy but it is associated with an increase in bleeding complications.^[16]^ Urapidil and dihydralazine have both been proven to be effective in lowering blood pressure in pregnant women with pre-eclampsia. The data are still accumulating, but current evidence suggests that sumatriptan does not carry a risk to either the fetus or the mother. With discrepancies on the current literature, the main aim of the study is to determine how migraine OTC painkillers can affect the hypertensive disorders in pregnant women.

## 2 Research question

What is the effect of migraine OTC painkillers on the hypertensive disorders of pregnancy?

## 3 Objectives

a. To evaluate the risk of hypertensive disorders in pregnant women using OTC painkillers to treat acute migraines vs women using OTCs to treat chronic migraines.
b. To outline migraine OTCs associated with low risk pregnancy outcomes vs migraine OTCs associated with high risk outcomes.
c. To assess what gestational age is safe for consumption of migraine OTC painkillers for both the fetus and the mother during pregnancy.

## 4 Methodology

This will be a systematic review and meta-analysis of published studies. This protocol is written in line with the recommendations of the preferred reporting items for systematic reviews and meta-analysis for protocols (PRISMA-P) guidelines 2015.^[17]^ The results will be reported based on the PRISMA 2015,^[17]^ statement and article screening and selection process will also be demonstrated through a PRISMA-P flow diagram. Furthermore, the current protocol has been registered with the international prospective register of systematic reviews: CRD42021232232.

## 5 Eligibility

### 5.1 Study design

This systematic review and meta-analysis will include randomized control trials, cohort, matched cohort, and cross-sectional studies with a clearly defined population, and interventions used. While, observational studies, reviews, case studies, and animal studies will be excluded in this study.

### 5.2 Participants

Migraine pregnant women.

### 5.3 Intervention

OTC painkillers.

### 5.4 Comparator

Migraineurs pregnant women.

### 5.5 Outcomes

Hypertensive disorders of pregnancy, eclampsia, pre-eclampsia.

### 5.6 Inclusions criteria

a. Evidence published in the English language.
b. Evidence published between the year 2011 and 2021.
c. Evidence from published global Randomized control trial, Matched cohort, cohort and cross sectional studies on the effect of migraine OTC painkillers on hypertensive disorders of pregnancy.
d. All of the criteria defining the impact of migraine OTC on the incidence of hypertensive disorders of pregnancy.

### 5.7 Exclusions criteria

a. Study which does not have the outcomes of interest as objectives.
b. Case reports, expert opinions and review/meta-analysis.
c. Evidence from migraine OTC to nonpregnant women will be excluded because the impact of migraine OTC is expected to be evaluated in pregnant women.

### 5.8 Search strategy

The following databases will be searched for eligible studies: Science direct, Medline, Embase, Pubmed, Africa Wide, Google scholar, ResearchGate, EBSCOhost, Web of Science, and the Cochrane Library, and LILACS. Medical subject headings (MeSH) such as “(((((((((((“migraine disorders”[MeSH Terms] OR (“migraine”[All Fields] AND “disorders”[All Fields]) OR “migraine disorders”[All Fields] OR “migraine”[All Fields]) AND (“pregnant women”[MeSH Terms] OR (“pregnant”[All Fields] AND “women”[All Fields]) OR “pregnant women”[All Fields])) AND ((“nonprescription drugs”[MeSH Terms] OR (“nonprescription”[All Fields] AND “drugs”[All Fields]) OR “nonprescription drugs”[All Fields] OR (“over”[All Fields] AND “counter”[All Fields]) OR “over the counter”[All Fields]) AND (“analgesics”[All Fields] OR “analgesics”[MeSH Terms] OR “analgesics”[All Fields] OR “painkillers”[All Fields]))) AND (migraineurs[All Fields] AND (“pregnant women”[MeSH Terms] OR (“pregnant”[All Fields] AND “women”[All Fields]) OR “pregnant women”[All Fields]))) AND (hypertensive[All Fields] AND (“disease”[MeSH Terms] OR “disease”[All Fields] OR “disorders”[All Fields]) AND (“pregnancy”[MeSH Terms] OR “pregnancy”[All Fields]))) OR (“pre-eclampsia”[MeSH Terms] OR “pre-eclampsia”[All Fields] OR (“pre”[All Fields] AND “eclampsia”[All Fields]) OR “pre eclampsia”[All Fields])) OR (“eclampsia”[MeSH Terms] OR “eclampsia”[All Fields])) OR (“hypertension, pregnancy-induced”[MeSH Terms] OR (“hypertension”[All Fields] AND “pregnancy-induced”[All Fields]) OR “pregnancy-induced hypertension”[All Fields] OR (“hypertension”[All Fields] AND “pregnancy”[All Fields]) OR “hypertension in pregnancy”[All Fields])) AND (“randomized controlled trial”[All Fields] OR “randomized controlled trials as topic”[MeSH Terms] OR “randomised controlled trial”[All Fields] OR “randomized controlled trial”[All Fields])) OR (“cohort studies”[MeSH Terms] OR (“cohort”[All Fields] AND “studies”[All Fields]) OR “cohort studies”[All Fields])) OR (matched[All Fields] AND (“cohort studies”[MeSH Terms] OR (“cohort”[All Fields] AND “studies”[All Fields]) OR “cohort studies”[All Fields] OR (“cohort”[All Fields] AND “study”[All Fields]) OR “cohort study”[All Fields]))) AND (“cross-sectional studies”[MeSH Terms] OR (“cross-sectional”[All Fields] AND “studies”[All Fields]) OR “cross-sectional studies”[All Fields] OR (“cross”[All Fields] AND “sectional”[All Fields] AND “study”[All Fields]) OR “cross sectional study”[All Fields])”])) and free text searches will be used to search the eligible articles which will be saved to the citation manager Zotero v5.0.81 (Zotero.org Virginia, USA). This software will also be used to remove duplicates. The title and abstracts of the articles remaining after exclusion of duplicates will be assessed for eligibility according to the inclusion and exclusion criteria.

### 5.9 Study selection

The full text of all potentially eligible studies will then be reviewed by 2 independent reviewers (SLN and WNP), and any disagreement between reviewers with respect to eligible studies for inclusion in the analysis will be assessed for more eligible studies. Initially, studies will be screened by the titles, abstracts, keywords, and synonyms then followed by the identification of the full-text articles. Should discrepancies arise between 2 authors (SLN, WNP), a third author will screen such studies, and consensus will be reached through discussion. Zotero v5.0.81 (Zotero.org) will be used to manage extracted data items, including saving relevant and excluded studies with reasons. Importantly, reference lists of included studies will be screened to confirm that no relevant studies are left out. Studies meeting the inclusion criteria will then be subjected to data collection, critical appraisal, risk, and quality evaluation.

## 6 Data management

### 6.1 Data collection process

The reviewers (SLN and WNP) will develop a data extraction form that will be used in the collection relevant data items. To reduce data entry errors, selected studies will be independently assessed by 2 reviewers (SLN and WNP), the third reviewer will be consulted for arbitration in case of any disagreements.

### 6.2 Data items

Extracted data items will include the author's name, year of publication, gestational age, type of OCT, dosage, adverse maternal outcomes: hypertensive disorders of pregnancy, eclampsia, pre-eclampsia,

### 6.3 Risk of bias in individual studies

To evaluate the potential risk of bias in randomized control trials, cohort, and matched cohort, Cochrane collaboration tool for assessing bias^[18]^ and Downs and Black checklist^[19]^ will be used. Two independent reviewers (SLN and WNP) will appraise all included studies and a third reviewer will be consulted in cases of disagreements.

### 6.4 Data synthesis

A summary of findings table will be used to provide a synthesis of the main outcomes of included studies. Data will be analyzed with Rev Manager (Version 5.3; Cochrane, from Carlifonia, USA) to conduct a meta-analysis. To measure statistical heterogeneity between studies, I^2^ and Chi squared statistical tests will be used.^[20,21]^ An I^2^ value of >50% will be considered substantial heterogeneity.^[22]^ To find the sources of heterogeneity within the included studies, a subgroup analysis and meta-regression comparing the study estimates from different study-level characteristics, quality, intervention type, and the reported effect measure of adverse events will be conducted.

## 7 Quality assessment of the cumulative evidence

The grading of recommendations, assessment, development and evaluation assessment tool^[23]^ will be used to assess the overall quality of evidence. Moreover, the quality of each included study will be independently evaluated by 2 authors (SLN, WNP). The third author will adjudicate in cases of disagreements. The quality of evidence will be assessed based on several factors such as study limitations, indirectness of results, and publication or reporting bias. The evidence of each outcome will be rated as high, moderate, low, or very low.

## 8 Discussion

This is a systematic review study that examines the potential effects of different types of migraine OTC painkillers on the hypertensive disorders of pregnancy. Some women experience migraine for the first time during pregnancy and some experience an increase in migraine symptoms especially during the first trimester.^[24]^ The appearance or worsening of migraine in pregnant women should be taken very seriously. These studies show that migraine symptoms, when accompanied by high blood pressure, can increase the risk of developing preeclampsia, eclampsia, hypertension or other vascular complications.^[25]^ Women whose migraine symptoms don’t decrease during pregnancy should be particularly vigilant.

The types of OTC medications that have been reported to be used mostly for migraines by pregnant women include paracetamol, acetaminophen, aspirin, and NSAIDs.^[26]^ This study is therefore undertaken to provide advice that needs to be tailored on the effects of these medications on maternal and fetal outcomes, however it may be hindered by 2 factors; the lack of safety data of these drugs in pregnancy as well as the duration of how long they should be consumed inspite the progression of the migraine.^[27]^ Prescribing is further complicated by both the mother's and the foetus’ changing physiologies as risk–benefit assessments alter throughout pregnancy. Migraine OTC painkillers treatment decisions may require the input of multi-disciplinary teams that consider the severity of the mother's migraine, maternal physiology, the drug's pharmacokinetic, pharmacodynamic and safety profile, and the developmental stage of the foetus. Unfortunately, there is a relative lack of high-quality randomized trials in the field of hypertension in pregnancy compared with studies in essential hypertension outside of pregnancy, and greater funding and uptake of collaborative research is still required in this field.^[28]^

Although generally safe, all migraine OTC painkillers are associated with some harm, particularly when recommended dosing limits are exceeded.^[29]^ The evidence base for making recommendations on the dosages of these medications for pregnant women remains limited. Since appropriate dose-finding studies may require large sample sizes (which may not be feasible), an indirect meta-analysis technique is suggested as a secondary research approach to see whether it can be helpful in the evaluation of dosages.^[30]^

## Author contributions

SLN and WNP conceptualized, designed, drafted and approved the final manuscript.

**Conceptualization:** Wendy Phoswa, Shalati L Nkuna.

**Methodology:** Wendy Phoswa.

**Resources:** Wendy Phoswa, Shalati L Nkuna.

**Supervision:** Wendy Phoswa.

**Validation:** Wendy Phoswa, Shalati L Nkuna.

**Writing – original draft:** Wendy Phoswa, Shalati L Nkuna.

**Writing – review & editing:** Wendy Phoswa, Shalati L Nkuna.
